# Editorial: Thyroid endocrine disruptors

**DOI:** 10.3389/fendo.2025.1554563

**Published:** 2025-01-29

**Authors:** Leonidas H. Duntas, Luca Chiovato

**Affiliations:** ^1^ Evgenideion Hospital, Unit of Endocrinology, Diabetes and Metabolism, National and Kapodistrian University of Athens, Athens, Greece; ^2^ Unit of Endocrinology and Metabolism, Laboratory for Endocrine Disruptors, Istituti Clinici Scientifici Maugeri IRCCS, Pavia, Italy

**Keywords:** endocrine disruptors, thyroid function, per-and polyfluoroalkyl substances (PFAS), selenomethionine, helicobacter pylori

Assembling a Research Topic is a well-known method of compiling a thesis based on published work. Another means is to gather several research papers highlighting a specific scientific area and include an introductory section and a summary of corresponding chapters.

Eight papers over the last 3 years in *Frontiers of Endocrinology* have been collected and summarized in this Editorial to heighten collective awareness of the impact of toxic environmental elements, mainly endocrine chemical disruptors, on thyroid function and disease pathogenesis.

Xiao et al. prospectively investigated parathyroid hormone (PTH), serum calcium, phosphorus, and 25-hydroxyvitamin D (25-OH-VD) levels at different time points before and after radioactive iodine (RAI) treatment in 259 differentiated thyroid carcinoma (DTC) patients. PTH, serum calcium, and phosphorus levels decreased in 5 days post-RAI, while about 20% developed clinically manifested hypocalcemia. Multivariate regression analysis revealed that baseline pre-RAI serum calcium < 2.27 mmol/L, PTH < 4.18 pmol/L, and negative 99mTcO4 − thyroid scintigraphy were risk factors for hypocalcemia 5 days post-RAI. It thus appears that β-rays released by RAI treatment for thyroid disease may disrupt parathyroid function.

Zhao et al. studied the metabolism of CD4+ T cells in 30 Hashimoto’s thyroiditis patients and 30 healthy controls. They observed an increased extracellular acidification rate (ECAR) and oxygen consumption rate (OCR) in the CD4+T cells, whereas in a murine model of spontaneous autoimmune thyroiditis (SAT), an elevation of the mTOR/HIF-1a/HK2/glycolysis pathway in CD4+ T cells was found. Treatment with the glucose blocker 2-deoxyD-glucose (2DG) and/or the mitochondrial complex 1 inhibitor, metformin could potentially modify the relationship between abnormally activated mTOR/HIF-1a/glycolysis and imbalanced CD4+ T cells subtype by decreasing the ratio of Th17 and Th1 T cells. This study presents novel pathogenetic aspects of HT and potential mechanisms of metformin action in thyroiditis.

Homburg et al. systematically reviewed the effects of triclosan, an antibacterial agent, on thyroid function. A total of 17 articles were analyzed of which 13 studies were observational and four interventional. Some studies showed a negative association of triclosan with T3 and T4 and a positive association with TSH, others showed the opposite, and the rest found no association. The authors thus concluded that due to the limited data, more interventional and well-controlled studies are needed to determine whether triclosan affects thyroid function.

Coperchini et al. studied the effects of per- and polyfluoroalkyl substances (PFAS), a group of synthetic compounds widely used in industry plants due to their low grade of degradation and flame resistance, on thyroid function. These characteristics are potentially dangerous for both human health and the environment. PFAS are persistent pollutants accumulating in water and soil that may be found in foods given that the chemicals are often used in disposable food packaging. Due to their persistence and potential harm to human health, some old-generation PFAS have been replaced by newly synthesized, allegedly safer PFAS. Even though exposure to PFAS in the environment remains a matter of concern worldwide, data on the thyroid-disrupting effect of these chemicals and on their impact on human health at different ages remain controversial.

Benvenga et al. examined the effects of myo-inositol (MI), seleno-L-methionine (Se), combined cMI + Se, and resveratrol on C cells of mice exposed to cadmium chloride (Cd Cl2). Previous studies showed that CdCl2 may induce hyperplasia and hypertrophy of C cells. C57 BL/6J adult male mice were studied for 14 days. Se at either 0.2 or 0.4 mg/kg/day failed to significantly increase follicular mean diameter while mildly decreasing calcitonin (Ct) positive cell number. In contrast, MI alone significantly increased follicular mean diameter number and substantially decreased CT-positive cell number, area, and cytoplasmic density. MI + Se 0.2 mg/kg/day or MI + Se 0.4 mg/kg/day administration appreciably improved all five indices. Furthermore, it has been hypothesized that the action of Se-dependent antioxidant enzymes may protect against Cd toxicity, thus reducing the oxidative stress and counteracting the apoptosis induced by endoplasmic reticulum stress.

Although iodine 131 (^131^I) is the main therapeutic tool for distant metastases of differentiated thyroid cancer, ^131^I overtreatment exerts many disrupting effects on several organ systems. However, Sa et al. found that two parameters can be used to predict the need for further ^131^I treatments, namely, Delta Tg % (pre-therapeutic Tg-post-therapeutic Tg/pre-therapeutic Tg × 100%) and maximum target/background ratio (T/Bmax) in each Rx (post ^313^I)-WBS. Using these parameters ^131I^ overtreatment can be avoided.

Wang et al. investigated whether helicobacter pylori (H-pylori) infection has a thyroid-disrupting effect. They found that H. pylori seropositive subjects had higher serum TSH than those with H. pylori seronegative. In stratified analyses, the adjusted association of serum TSH with H. pylori seropositivity was statistically significant in male, normal BMI, overweight, obese, and over 60-year-old subjects.

In their study, Chen et al. investigated thyroid function a year after percutaneous coronary intervention (PCI). PCI treatments result in the absorption of large volumes of iodinated contrast media (ICM) by administering ICM doses ranging from 80 to 530 mL. The main results were the following: (i) the serum level of FT4 was significantly increased; (ii) the volume of ICM correlated with composite endpoints and hyperthyroidism with a nonlinear dose-response relationship; and (iii) the risk of hyperthyroidism increased in the high-volume ICM patient group. This indicates the need to check thyroid function in PCI-treated patients carefully.

## In practice

Several compounds, including synthetic substances, metals, contrast media, and radioiodine, are strongly suspected of being endocrine disruptors that affect thyroid and parathyroid function and thereby induce disease. However, no large studies exist to date demonstrating that the studied chemicals and metals exert thyroid disruption. The results of the above-presented recent and important papers are anticipated to increase awareness of the multiple risks of EDC exposure to thyroid health among researchers and the public.

